# Canine Leishmaniasis in Southeastern Spain

**DOI:** 10.3201/eid1505.080969

**Published:** 2009-05

**Authors:** Joaquina Martín-Sánchez, Manuel Morales-Yuste, Carmen Acedo-Sánchez, Sergio Barón, Victoriano Díaz, Francisco Morillas-Márquez

**Affiliations:** Universidad de Granada, Granada, Spain (J. Martín-Sánchez, M. Morales-Yuste, S. Barón, V. Díaz, F. Morillas-Márquez); Laboratorio de Análisis Veterinarios ANLAVE, Granada (C. Acedo-Sánchez)

**Keywords:** Vector-borne infections, canine leishmaniasis, *Leishmania infantum*, global change, risk factors, Spain, dispatch

## Abstract

To examine prevalence changes and risk factors for canine leishmaniasis, we conducted a cross-sectional seroprevalence study and a survey during April–June 2006. Seroprevalence had increased at the meso-Mediterranean bioclimatic level over 22 years. Risk was highest for dogs that were older, large, lived outside, and lived at the meso-Mediterranean level.

It has been suggested that climate change has the potential to change the transmission intensity of vector-borne diseases such as leishmaniasis, but supporting literature is lacking (*1*,*2*). Because long-term quality data on leishmaniasis caused by *Leishmania infantum* and its vector (*3*–*9*) are available for the Alpujarras region of southeastern Spain (Figure 1), this is an ideal area for studying changes in the prevalence of canine leishmaniasis in a changing environment. Our study objectives were to determine whether any changes had occurred in the prevalence of canine leishmaniasis over 22 years and to identify risk factors for this disease.

**Figure 1:**
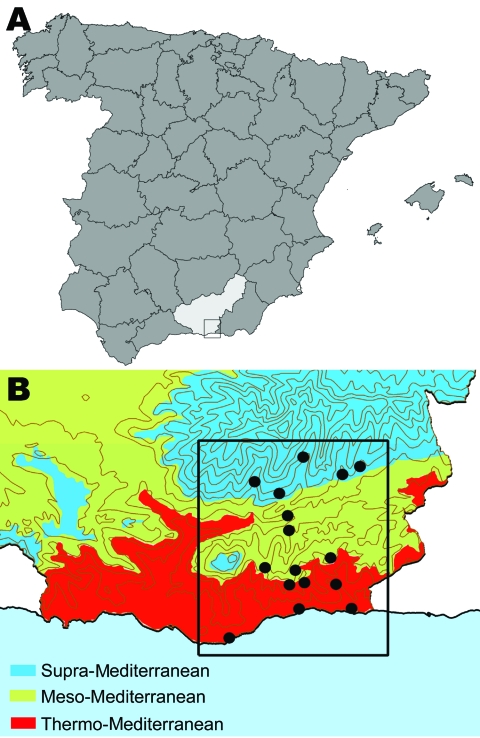
A) Location of the Alpujarras in southeastern Spain (37º00’–37º20’N and 3º00’–3º30’W). B) Bioclimatic levels (shading) and villages (black dots) where serum samples were collected from dogs to examine for leishmaniasis prevalence and sandflies were collected to estimate densities, April–June 2006. Of 1,675 sandflies captured, 269 were identified by morphologic appearance as *Phlebotomus perniciosus* (density 0–165 specimens/m^2^) and 22 as *P. ariasi* (0 and 11 specimens/m^2^).

## The Study

To achieve the first objective, we conducted a cross-sectional study in the Alpujarras from April through June 2006. We then compared current leishmaniasis seroprevalence data with data from 2 cross-sectional surveys conducted in 1984 and 1991 (*3*–*5*). The villages sampled for all 3 studies were similar and had been selected at random from within each of the 3 bioclimatic levels (thermo-, meso-, and supra-Mediterranean) that comprise the inhabited zone of the Alpujarras (Figure 1) (*10*). For each level, respectively, altitudes are 0–700, 600–900, and 900–1,800 m above sea level; annual mean temperatures are 17–19, 13–17, and 8–15°C; and annual rainfalls are 200–350, 600–1,000, and 1,000–1,600 inches. The dates for sample collection were set to coincide with organized events at which dogs were gathered (e.g., antirabies vaccination campaigns). All 3 surveys used indirect immunofluorescence for diagnosis; protocols and positivity threshold were identical. Dogs with a titer >160 were considered positive. To determine and compare the existence or lack of statistically significant differences between present and past prevalence rates, we used the χ^2^ or Fisher exact test.

To achieve the second objective, we conducted a survey. The owners of the dogs included in the cross-sectional study conducted in the Alpujarras from April to June 2006 were asked to complete an epidemiologic record for each dog tested; data on the animal and its environment were recorded for subsequent use in univariate and multivariate logistic regression analyses (Tables 1, 2). Density data were included for 2 vectors, *Phlebotomus perniciosus* and *P. ariasi* sandflies, captured with sticky traps in June 2006 in the same villages in which the surveys were conducted. No retrospective entomologic analysis was performed as had been done for canine leishmaniasis.

**Table 1:** Possible factors associated with canine leishmaniasis, southeastern Spain*

Variables	No. dogs	% Dogs with canine leishmaniasis	Relative likelihood	p value
Bioclimatic level	439	13.0	–	0.005
Thermo-Mediterranean	210	13.3	Ref	–
Meso-Mediterranean	139	20.1	1.640	0.092
Supra-Mediterranean	90	1.1	0.073	0.011
Habitat	438	13.0	–	0.999
Rural	435	13.1	Ref	–
Urban/peri-urban	3	0.0	0.000	–
Sex	435	12.4	0.662	0.178
Male	253	14.2		
Female	182	9.9		
Age, y	421	13.0	2.094	<0.001
<4	232	9.5		
>4	189	18.0		
Weight, kg	405	13.6	1.859	0.048
<25	316	11.9		
>25	89	20.0		
Fur length	349	15.5	0.494	0.069
Short/ medium	255	17.6		
Long	94	9.6		
Activity	439	13.0	–	<0.001
Pet	258	8.5	Ref	–
Hunting	133	15.0	1.899	0.052
Other†	48	31.3	4.876	<0.001
Location during daytime	373	15.3	–	<0.001
House	151	4.0	Ref	–
Outside	122	28.7	9.722	<0.001
In kennels	100	16.0	4.603	0.002
Location at night	435	13.1	0.384	0.001
Outdoors	186	19.4		
Indoors	249	8.4		
Travel away from home	438	13.0	1.384	0.461
No	396	12.6		
Yes	42	16.7		
Clinical signs of leishmaniasis	439	13.0	2.129	0.122
No	413	12.3		
Yes	26	23.1		
Fly protection	375	15.2	1.551	0.969
No	361	15.0		
Yes	14	21.4		
*Phlebotomus perniciosus* density	439	13.0	–	0.005
<4 sandflies/m^2^	303	9.9	Ref	
>4 sandflies/m^2^	136	19.9	2.254	
*P. ariasi* density	439	13.0	–	<0.001
<6 sandflies/m^2^	383	10.7	Ref	
>6 sandflies/m^2^	56	28.6	3.337	

*Univariate analysis by logistic regression. Dogs considered to have canine leishmaniasis were those with antibody titer >160. We investigated the existence of interaction and/or confusion between variables by constructing and comparing logistic regression models. The statistical analysis was performed using the software package SPSS 15.0 (www.spss.com). Confusion was noted between the location during daytime and location at night, so location during daytime was excluded from the multivariate analysis. No interaction was detected between any pair of independent variables. Ref, referent. †Guard dogs (n = 34), sheepdogs (n = 8), stray dogs (n = 4), dogs in kennel (n = 2).

**Table 2:** Factors associated with canine leishmaniasis, southeastern Spain, final model*

Variables (no. dogs)	OR (95% CI)	p value
Bioclimatic level	–	0.001
Thermo-Mediterranean (189)	–	–
Meso-Mediterranean (134)	0.538 (0.196–1.476)	0.228
Supra-Mediterranean (82)	0.013 (0.001–0.126)	<0.001
Age, y	–	0.001
<4 (224)	–	
>4 (181)	3.223 (1.604–6.474)	
Weight, kg	–	0.069
<25 (316)	–	
>25 (89)	1.985 (0.948–4.156)	
Activity	–	0.00
Pet (232)	–	–
Hunting (129)	2.401 (1.060–5.442)	0.036
Other (44)†	4.831 (1.909–12.226)	0.001
Location at night	–	<0.001
Indoors (229)	–	
Outdoors (176)	3.304 (1.704–6.406)	
*Phlebotomus perniciosus* density	–	<0.001
<4 sandflies/m^2^ (277)	–	
>4 sandflies/m^2^ (128)	7.029 (2.632–18.769)	

*Determined by multivariate logistic regression. With age as continuous variable (0.1–25 years, average 4.7 years), odds ratio (OR) = 1.142; with weight as continuous variable (2–60 kg, average = 17.7), OR = 1.047. CI, confidence interval. †Guard dogs, sheepdogs, stray dogs, and dogs in kennels.

**Figure 2:**
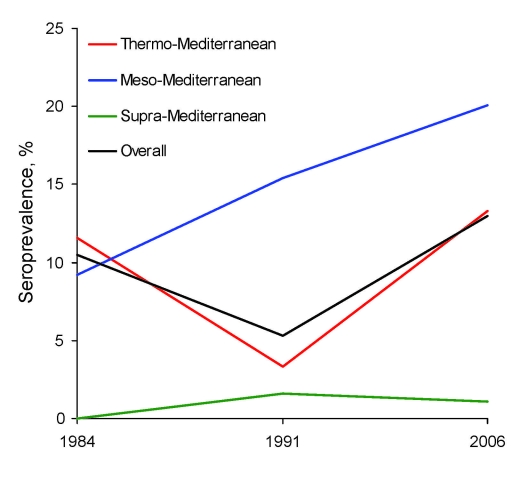
Canine leishmaniasis seroprevalence rates in the Alpujarras, Spain, 1984–2006, by time and bioclimatic level.

In our 2006 survey, 57 (13.0%) of 439 dogs had an antibody titer >160 (seroprevalence rate), 268 (61.0%) dogs had titers 20–80, and 114 (26.0%) had no titer. In terms of bioclimatic level, canine leishmaniasis seroprevalence was 20.1% at the meso-Mediterranean, 13.3% at the thermo-Mediterranean, and only 1.1% at the supra-Mediterranean levels. Statistically significant differences (χ^2^ test p<0.001) indicate that these differences are not random. The evolution-in-time analysis (Figure 2) shows how over 22 years (1984–2006), seroprevalence of canine leishmaniasis has progressively increased at the meso-Mediterranean level, climbing from 9.2% in 1984 to 15.4% in 1991 and finally to 20.1% in 2006 (p = 0.015); in contrast, no significant changes have taken place in global prevalence or in the other 2 bioclimatic levels studied (*3–5*; this study).

Dogs at greatest risk for canine leishmaniasis in the disease-endemic region of the Alpujarras were large dogs (>25 kg) and older dogs (>4 years) that worked as guard dogs or sheepdogs, slept outdoors, and lived at the thermo- or meso-Mediterranean level in a village such as Torvizcón, where the *P. perniciosus* density is >4 sandflies/m^2^. Risk for these dogs was 54,571× greater than for dogs that were kept as pets, were small, were <4 years of age, and slept inside a house in a village such as Mecina Bombarón or Pórtugos at the supra-Mediterranean level where *P. perniciosus* density is <4 sandflies/m^2^.

## Conclusions

Among the drivers of global change that have the potential to influence vector-borne diseases, climatic and nonclimatic (socioeconomic, demographic, and environmental) factors have been cited (*1*,*2*). Although it is not easy to attribute our findings—progressive increase in seroprevalence at the meso-Mediterranean level and drop and subsequent rise at the thermo-Mediterranean level (Figure 2)—to 1 or more drivers, we can attempt to find some form of association in the changes that have occurred in the Alpujarras throughout these 2 decades. Studies on climate change in Spain confirm a warming tendency (reflected at the global level), which provides evidence that temperatures have been increasing for a quarter of a century (*11*,*12*). Thus, we must assume that changes in temperature, rainfall, or humidity will have equally affected the 3 bioclimatic levels researched in the Alpujarras and may have influenced the spatial and temporal distribution and the seasonal dynamics of sandflies. An increase was detected in the period of *P. perniciosus* activity in the region; these effects were probably more notable at the meso-Mediterranean level because this is where the density of this vector species is at its highest (*6*; Martín-Sánchez et al., unpub. data). During the time period researched, the human population remained constant in the Alpujarras; it has, however, progressively decreased in the disperse populations and increased in population centers. Unfortunately, no official figures for the canine population are available, although we were informed by staff of local town halls that in 1991, the 615 dogs analyzed accounted for ≈100% of the total number of dogs registered in the villages sampled (*3*). The Alpujarras economy is based mainly on agriculture and livestock. One change during the period studied was the gradual disappearance of livestock enclosures in rural population centers. These enclosures appear to create a substantial risk for infection with *Leishmania* spp. (*13*,*14*) and are ideal places for sandfly blood sucking, mating, and oviposition (*15*).

With respect to risk factors, the increase in canine leishmaniasis seroprevalence as animal’s age increases seems logical because in a leishmaniasis-endemic area, the greater the age, the longer the animal will have been exposed to sandflies and the greater the probability of having been bitten by an infected female sandfly. Seroprevalence of canine leishmaniasis also increases gradually with weight, which could be attributed to the vector being more attracted to larger animals. Dogs that sleep outdoors are at greater risk than those sleeping indoors because the density of the vector (*P. perniciosus*) is greater outdoors than inside a house. The association between *P. perniciosus* density and canine leishmaniasis seems logical, considering that *P. perniciosus* is the main vector species in the Alpujarras. This is the only species found to have been infected in this region (*8*,*9*), although *P. ariasi* is a proven vector in the nearby region of the Axarquia (*13*).

In the Alpujarras, the percentage of dogs kept as pets has increased from 42% in 1991 (*3*) to 59% in 2006 (p<0.001). This increase corresponds to a general increase in standard of living and, along with other factors such as the disappearance of livestock enclosures from within population centers, would have acted to reduce transmission. Only the extension of the vector’s activity period, which was detected at some trap sites, would have acted to increase transmission. Such extension of activity may be related to the increase in temperature brought about by climate change (*11*,*12*).
